# The Suppressive Activity of Fucofuroeckol-A Derived from Brown Algal *Ecklonia stolonifera* Okamura on UVB-Induced Mast Cell Degranulation

**DOI:** 10.3390/md16010001

**Published:** 2018-01-04

**Authors:** Thanh Sang Vo, Se-Kwon Kim, BoMi Ryu, Dai Hung Ngo, Na-Young Yoon, Long Giang Bach, Nguyen Thi Nhat Hang, Dai Nghiep Ngo

**Affiliations:** 1 NTT Institute of Hi-Technology, Nguyen Tat Thanh University, Ho Chi Minh City 700000, Vietnam; vtsang@ntt.edu.vn; 2 Department of Marine Life Science, College of Ocean Science and Technology, Korea Maritime and Ocean University, Busan 606-791, Korea; 3 School of Pharmacy, the University of Queensland, Brisbane QLD 4072, Australia; ryu.bomi@gmail.com; 4 Faculty of Natural Sciences, Thu Dau Mot University, Thu Dau Mot City 820000, Binh Duong Province, Vietnam; hungdaingo83@yahoo.com (D.H.N.); hangntn@tdmu.edu.vn (N.T.N.H.); 5 Food and Safety Research Center, National Fisheries Research & Development, Busan 46083, Korea; dbssud@hanmail.net; 6 Department of Science and Technology, Nguyen Tat Thanh University, Ho Chi Minh City 700000, Vietnam; blgiang@ntt.edu.vn; 7 Faculty of Chemistry, University of Science-VNU-HCM City, 227 Nguyen Van Cu Street, Ho Chi Minh City 700000, Vietnam; 8 Department of Biochemistry, Faculty of Biology and Biotechnology, University of Science, Vietnam National University, Ho Chi Minh City 700000, Vietnam

**Keywords:** *Ecklonia stolonifera*, phlorotannin, Fucofuroeckol-A, anti-allergy, degranulation, mast cells

## Abstract

UV light, especially UVB, is known as a trigger of allergic reaction, leading to mast cell degranulation and histamine release. In this study, phlorotannin Fucofuroeckol-A (F-A) derived from brown algal *Ecklonia stolonifera* Okamura was evaluated for its protective capability against UVB-induced allergic reaction in RBL-2H3 mast cells. It was revealed that F-A significantly suppress mast cell degranulation via decreasing histamine release as well as intracellular Ca^2+^ elevation at the concentration of 50 μM. Moreover, the inhibitory effect of F-A on IL-1β and TNF-α productions was also evidenced. Notably, the protective activity of F-A against mast cell degranulation was found due to scavenging ROS production. Accordingly, F-A from brown algal *E. stolonifera* was suggested to be promising candidate for its protective capability against UVB-induced allergic reaction.

## 1. Introduction

Sunlight is a continuous spectrum of electromagnetic radiation that is divided into three major spectrums of wavelength such as ultraviolet, visible, and infrared [1]. The UV range is the most significant spectrum of sunlight with a wavelength from 10 nm to 400 nm, shorter than that of visible light but longer than X-rays. Ultraviolet (UV) radiation is divided into three distinct bands including UVA (320–400 nm), UVB (290–320 nm), and UVC (200–290 nm) in order of decreasing wavelength and increasing energy [2]. UVC light is absorbed by the atmosphere, while approximately 90–99% of UVA and 1–10% of UVB reaches the earth’s surface [3]. Different wavelengths and energy associated with UV subdivision correspond to distinctly different effects on living tissue. UV light causes various biological reactions including acute inflammation and cancer, as in sunburn and eruptions of the skin [4,5]. Notably, it has been reported that mast cell activation including histamine release is involved in the UV-induced sunburn reaction [6]. At a low dose, UVB light inhibits histamine release from mast cells induced by compound 48/80 [7], A23187 [8], and substance P [9]. However, UVB light causes histamine release from rat peritoneal mast cells at doses higher than 7.8 kJ/m^2^ [10]. Accordingly, UVB light can be applied as an allergic therapeutic at a low dose, whereas it is also a cause of allergic reaction from a high dose, such as exposure to outdoor sunlight. Thus, a protective agent that is able to block UVB from sunlight is necessary for humans when they go outside.

In recent years, seaweeds have served as an important source of bioactive natural substances that possess various pharmaceutical properties [11]. Among them, brown seaweed has been recognized as a rich source of phlorotannins, which are formed by the polymerization of phloroglucinol (1,3,5-tryhydroxybenzene) monomer units and biosynthesized through the acetate-malonate pathway [12]. Phlorotannins exhibit various beneficial bioactivities such as anti-oxidant, anti-cancer, anti-diabetic, anti-HIV, matrix metalloproteinase enzyme inhibition, and anti-hypertensive activities [13]. Notably, several phlorotannins from brown algae have been reported to be effective against allergic reaction in recent years. Dieckol, 6,6′-bieckol, and fucodiphloroethol G from *Ecklonia cava* were found to be strong inhibitors of histamine release from KU812 and RBL-2H3 cells with an IC_50_ range of 27.8–55.1 µM [14,15]. Likewise, dioxinodehydroeckol and phlorofucofuroeckol A from *E. stolonifera* have been demonstrated to suppress intracellular calcium elevation and histamine release from CRA-1-stimulated KU812 cells [16]. Although the anti-allergic activities of phlorotannins have been well-evidenced, their protective effect against UVB-induced allergic reactions has been not reported. Meanwhile, phlorotannins (dieckol) from *E. cava* has been known to possess strong protective activity against UV-B radiation-induced DNA damage. Moreover, it can reduce the intracellular reactive oxygen species generated by gammaray radiation [17]. Therefore, phlorotannins from brown seaweeds are suggested as effective protective agents against UVB-induced damages. Accordingly, the present study was designed to evaluate the protective effects of phlorotannin Fucofuroeckol-A derived from brown algal *Ecklonia stolonifera* Okamura against UVB-induced mast cell activation.

## 2. Results and Discussion

### 2.1. Structure Elucidation of Phlorotannin

**Figure 1:**
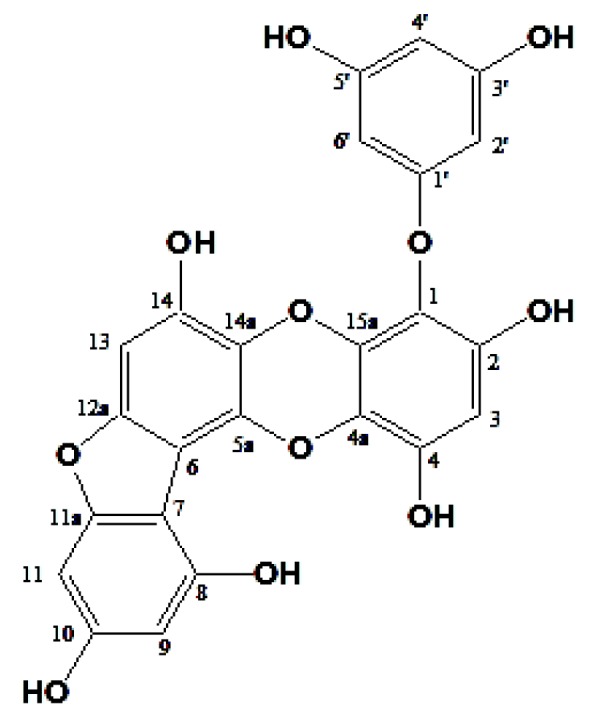
Chemical structures of Fucofuroeckol-A isolated from *E. stolonifera* Okamura.

Fucofuroeckol-A (F-A) was isolated as a pale brown powder. The molecular formula was established as C_24_H_14_O_11_. ^1^H-NMR (400 MHz, DMSO-*d*_6_) δ: 10.05 (1H, s, 14-OH), 9.88 (1H, s, 4-OH), 9.76 (1H, s, 10-OH), 9.44 (1H, s, 2-OH), 9.18 (2H, s, 3′, 5′-OH), 8.22 (1H, s, 8-OH), 6.71 (1H, s, H-13), 6.47 (1H, d, *J* = 1.1 Hz, H-11), 6.29 (1H, s, H-3), 6.25 (1H, d, *J* = 1.5 Hz, H-9), 5.83 (1H, s, H-4′), 5.76 (2H, d, *J* = 1.5 Hz, H-2′, 6′). Moreover, ^13^C-NMR (100 MHz, DMSO-*d*_6_) δ: 160.7 (C-1′), 158.8 (C-3′, 5′), 158.3 (C-11a), 157.6 (C-10), 150.5 (C-12a), 150.2 (C-8), 146.9 (C-2), 144.4 (C-14), 142.0 (C-4), 136.8 (C-15a), 133.6 (C-5a), 126.1 (C-14a), 122.6 (C-4a), 122.4 (C-1), 103.1 (C-6), 102.4 (C-7), 98.2 (C-3), 98.0 (C-9), 96.3 (C-4′), 94.6 (C-13), 93.7 (C-2′, 6′), 90.5 (C-11) (Figure 1).

### 2.2. Effect of F-A on Mast Cell Degranulation Induced by UVB

**Figure 2:**
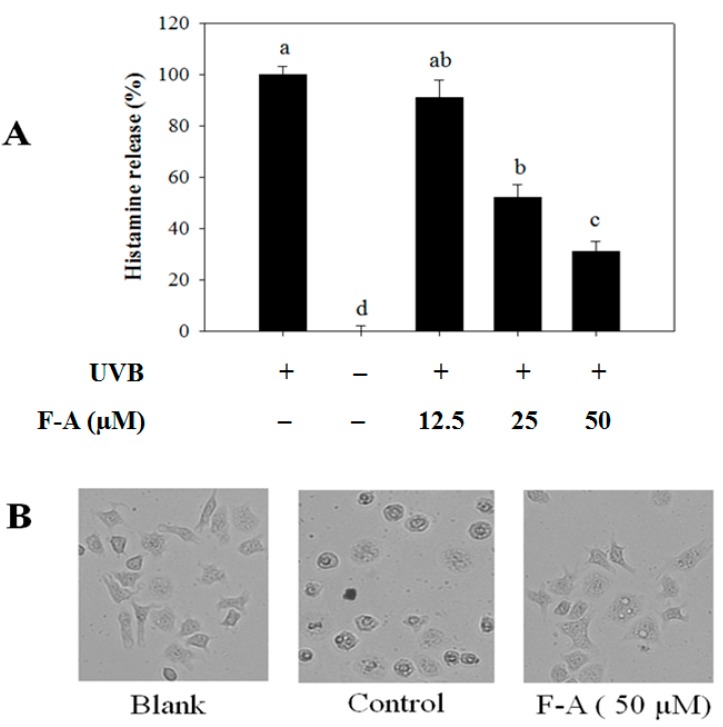
Effect of Fucofuroeckol-A (F-A) on mast cells degranulation in UVB-exposed RBL-2H3 cells. The cells were pretreated with F-A for 24 h before exposing to UVB for 60 min. (**A**) The levels of histamine release were measured via a spectrofluorometric assay. Each determination was made in three independent experiments, and the data are shown as means ± SD. Different letters a–d indicate significant difference among groups (*p* < 0.05) by Duncan’s multiple-range test; (**B**) The representative images of the cells were assessed by using light microscopy (magnification, ×20).

Although the reasons why allergies develop are not known, there are some substances that commonly cause an allergic reaction such as pet dander, bee stings, certain foods (nuts or shellfish), pollen, or molds [18]. Moreover, UV light, especially UVB, has also been reported to be able to trigger allergic reaction, leading to mast cell degranulation and histamine release [10]. Thus, compounds possessing protective activities against UVB light may influence its anti-allergic properties via the inhibition of mast cell degranulation and histamine release. Hence, the effect of F-A on mast cell degranulation was first evaluated by measuring histamine release induced by UVB. Figure 2 shows that F-A significantly decreases histamine release from the activated mast cells in a dose-dependent manner. The histamine release level upon pretreatment with 50 µM of F-A was 31%, as compared to the control group exposed to UVB alone (Figure 2A). On the other hand, its inhibitory effect on mast cell degranulation was also confirmed by testing cell morphological changes. In the normal condition, mast cells were generally branch-shaped with clear membranes, whereas the activated cells induced by UVB were round-shaped, and had reduced cell size, disrupted boundaries, and irregular surfaces. However, F-A-pretreated cells before being exposed to UVB exhibited a protective effect against the morphological changes (Figure 2B). This indicates that F-A is capable of protecting mast cells from UVB, thus blocking the mast cell degranulation and histamine release from the UVB-exposed mast cells.

### 2.3. Effect of F-A on Intracellular Ca^2+^ Elevation in UVB-Exposed RBL-2H3 Mast Cells

**Figure 3:**
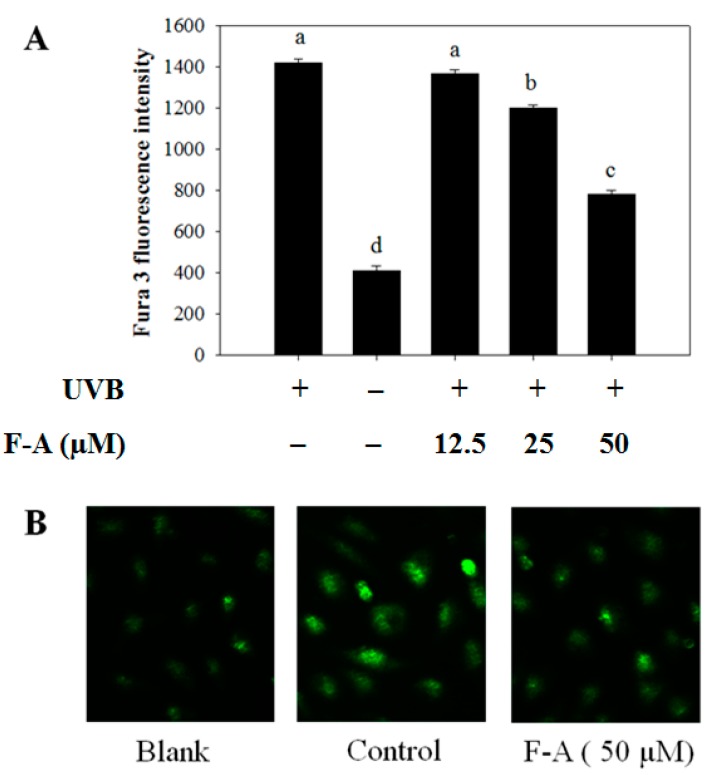
Effect of F-A on intracellular Ca^2+^ elevation in UVB-exposed RBL-2H3 mast cells. (**A**) The cells were pretreated with various doses of F-A for 24 h before incubating with Fura-3/AM for 60 min. The cells were then exposed to UVB for 10 min. The level of intracellular Ca^2+^ was monitored by a spectrofluorometric assay. Each determination was made in three independent experiments, and the data are shown as means ± SD. Different letters a–d indicate significant difference among groups (*p* < 0.05) by Duncan’s multiple-range test; (**B**) The representative images of the cells were assessed by using light microscopy (magnification, ×20).

The process of mast-cell degranulation requires the elevation of intracellular Ca^2+^ levels. Intracellular Ca^2+^ elevation is important in the regulation of granule-plasma membrane fusion [19]. The increase in intracellular Ca^2+^ concentration is a necessary and sufficient stimulus for mast-cell degranulation. Thus, we also examined whether F-A alleviates the intracellular Ca^2+^ level in UVB-exposed RBL-2H3 mast cells. Figure 3 shows that UVB induced the elevation of intracellular Ca^2+^ level in mast cells. Meanwhile, the pretreatment of F-A caused significant inhibition of the intracellular Ca^2+^ elevation. Notably, the inhibitory effect of intracellular Ca^2+^ elevation was observed to be effective at a concentration of 50 µM of F-A pretreatment (Figure 3A). Similarly, the fluorescence intensity in photographs, shown in Figure 3B, also indicated that F-A remarkably decreased the intracellular Ca^2+^ density in RBL-2H3 mast cells exposed to UVB. As a result, the alleviative effects of F-A on intracellular Ca^2+^ elevation resulted in the inhibition of granule-plasma membrane fusion, thus reducing mast cell degranulation and histamine release from the activated mast cells. It is supported by previous reports that some anti-allergic drugs inhibit histamine release via the inhibition of intracellular Ca^2+^ elevation [20,21].

### 2.4. Effect of F-A on Cytokine Production in UVB-Exposed RBL-2H3 Mast Cells

**Figure 4:**
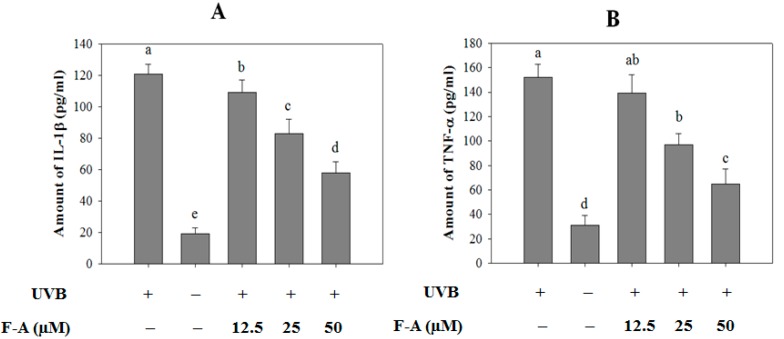
Effects of F-A on cytokine production in UVB-exposed RBL-2H3 mast cells. The cells were pretreated with various concentrations of F-A for 24 h before being exposed to UVB for 2 h. The production levels of IL-1β (**A**) and TNF-α (**B**) were quantified in culture media using commercial ELISA kits. Each determination was made in three independent experiments, and the data are shown as means ± SD. Different letters a–e indicate significant difference among groups (*p* < 0.05) by Duncan’s multiple-range test.

Besides histamine release, mast cell degranulation also leads to the production of several cytokines, such as IL-1β and TNF-α. The excessive expression and production of these cytokines alter the local microenvironment and eventually lead to the recruitment of inflammatory cells such as neutrophils and eosinophils [22]. Therefore, the modulation of inflammatory cytokines from mast cells is a one of the key indicators of reduced allergic symptoms. Herein, the production levels of IL-1β (Figure 4A) and TNF-α (Figure 4B) were observed to increase in the culture supernatants of UVB-exposed RBL-2H3 mast cells. The amounts of IL-1β and TNF-α from the exposed cells were 121 ± 6 and 152 ± 11 pg/mL, respectively, whereas the correlative amounts of these cytokines in the non-exposed cells were 19 ± 4 and 31 ± 8 pg/mL, respectively. Conversely, these increases were considerably diminished in a concentration-dependent manner by F-A pretreatment. At the concentration of 50 µM, F-A reduced IL-1β and TNF-α levels to 58 ± 7 and 65 ± 12 pg/mL, respectively.

### 2.5. Effect of F-A on ROS Production in UVB-Exposed RBL-2H3 Mast Cells

**Figure 5:**
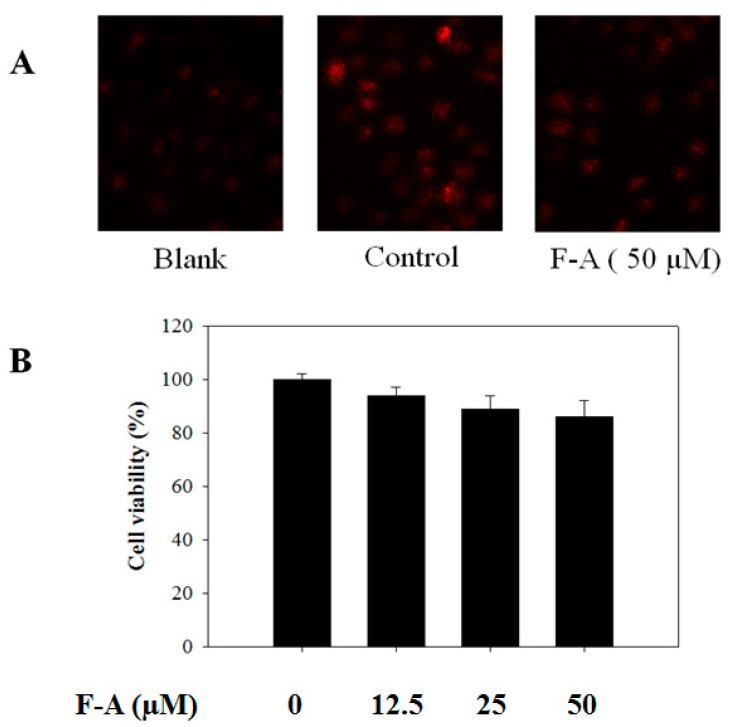
Effect of F-A on ROS production in UVB-exposed RBL-2H3 mast cells and cell viability. (**A**) The cells were pretreated with different doses of F-A for 24 h before being incubated with dihydroethidium for 60 min. The cells were then exposed to UVB for 60 min. The level of ROS production was monitored by a light microscope with 20× magnification; (**B**) The cells were pretreated with different concentrations of F-A for 24 h before being exposed to UVB for 2 h. Cell viability was demonstrated by the MTT method, and the results are expressed as a percentage of surviving cells over blank cells (no addition of F-A and UVB). Each determination was made in three independent experiments, and the data are shown as means ± SD.

More importantly, the role of ROS as an inducer of Ca^2+^ elevation and degranulation in mast cells has been reported in numerous recent studies [23,24]. Meanwhile, the inhibition of ROS production by diphenyleneiodonium (DPI) or antioxidants such as (−)-epigallocatechin gallate resulted in the suppression of IgE-mediated histamine release [25]. Thus, ROS production is a potential target for the downregulation of mast cell degranulation. Accordingly, we attempted to determine whether F-A blocks ROS production in the activated mast cells using a dihydroethidium fluorescence indicator. A light microscope assay showed that the UVB-exposed cells significantly increased the fluorescence density of ROS in the control group (exposure to UVB only). However, the fluorescence density of ROS was markedly decreased by F-A pretreatments at a concentration of 50 µM, indicating the inhibitory effect of F-A on ROS production in UVB-exposed mast cells (Figure 5A). Many researchers have shown that phenolic compounds from marine algae have strong antioxidant activities on free radicals and possess radio-protective activity against UVB light [17,26,27]. These results suggested that the effective antioxidant activity and radio-protective activity of F-A significantly contributes to the depression of mast cell degranulation.

To exclude the possibility that the inhibitory activities of F-A were due to cytotoxicity, an MTT assay was performed in RBL-2H3 cells pretreated with various concentrations of F-A for 24 h before exposure to UVB (20 kJ/m^2^) for 2 h. With the concentrations used in this study (12.5, 25, or 50 µM) and the exposure of UVB, none of the treatments affected cell viability (Figure 5B). Thus, the inhibitory activities of F-A on mast cell degranulation and ROS production were not due to any cytotoxic effect on RBL-2H3 cells.

## 3. Materials and Methods

### 3.1. Reagents and Materials

Fucofuroeckol-A (F-A) was kindly donated by Dr. Na-Young Yoon (Food and Safety Research Center, National Fisheries Research and Development, Busan, Korea). Enzyme immunoassay reagents for cytokine assays were purchased from R&D Systems (Minneapolis, MN, USA). Calcium-specific fluorescence probe (Fura-3/AM) was purchased from Santa Cruz Biotechnology Inc. (Santa Cruz, CA, USA). All other reagents were purchased from Sigma-Aldrich (St. Louis, MO, USA).

### 3.2. Cell Culture and Cell Viability Assay

RBL-2H3 mast cells were purchased from the Korean Cell Line Bank (Seoul, Korea). Cells were cultured in a humidified atmosphere containing 5% CO_2_ at 37 °C using Dulbecco’s Modified Eagle Medium (DMEM) supplemented with 10% heat-inactivated fetal bovine serum (FBS), 2 mM l-glutamine, 10 mM HEPES buffer, 100 U/mL of penicillin G, and 100 mg/mL of streptomycin.

The viability levels of RBL-2H3 cells was determined by MTT [3-(4,5-dimethyl-2-yl)-2,5-diphenyltetrazolium bromide] assay. The cells were grown in 96-well plates at a density of 2 × 10^5^ cells/mL. Then, cells were washed with fresh medium and pretreated with different concentrations of F-A for 24 h before being exposed to UVB (20 kJ/m^2^) for 2 h. Cells were rewashed and pretreated with MTT solution (1 mg/mL, final concentration) for 4 h. Finally, the supernatant was removed, and DMSO (100 µL) was added to solubilize the formed formazan salt. The amount of formazan salt was determined by measuring the absorbance at 540 nm using a microplate reader (GENios^®^ Tecan Austria GmbH, Grodig/Salzburg, Austria). Viability of cells was quantified as a percentage compared to blank.

### 3.3. Histamine Release Assay

RBL-2H3 cells were seeded into 24-well plates (2 × 10^5^ cells/mL). Cells were pretreated with different concentrations of F-A (12.5, 25, or 50 µM) for 24 h before being exposed to UVB (20 kJ/m^2^) for 60 min. Histamine release in the supernatants was determined as previously described [28]. Histamine release levels were calculated as a percentage compared to the control: Release ratio (%) = (T − B)/(C − B) × 100, where B is the group with neither stimulation nor the sample treatment, C is the stimulated group without treatment of the tested sample, and T is the stimulated group with the presence of the tested sample.

### 3.4. Measurement of the Intracellular Ca^2+^ Level

RBL-2H3 cells were seeded into black 96-well plates (2 × 10^5^ cells/mL) and pretreated with F-A (12.5, 25, or 50 µM) for 24 h before being incubated with Fura-3/AM (2 µM, final concentration) for 60 min. The cells were then washed with Tyrode buffer and exposed to UVB (20 kJ/m^2^) for 10 min. The Fura-3/AM fluorescence intensity was measured at an excitation wavelength of 360 nm and an emission wavelength of 528 nm using a microplate reader (GENios^®^ Tecan Austria GmbH, Grodig/Salzburg, Austria). In addition, fluorescence images were visualized and photographed under a fluorescence microscope (CTR 6000, Leica, Wetzlar, Germany) after the addition of 2% paraformaldehyde.

### 3.5. Measurement of Cytokine Production

RBL-2H3 cells were seeded into 24-well plates (2 × 10^5^ cells/mL) and pretreated with F-A (12.5, 25, or 50 µM) for 24 h before being exposed to UVB (20 kJ/m^2^) for 2 h. The supernatants were collected, and the production of IL-1β and TNF-α were quantified by sandwich immunoassays following the protocol of R&D systems.

### 3.6. Measurement of ROS Production

RBL-2H3 cells were seeded into 12-well plates (1 × 10^4^ cells/mL) and pretreated with F-A (12.5, 25, or 50 µM) for 24 h before being incubated with dihydroethidium (5 µM, final concentration) for 60 min at 37 °C. The cells were then washed with Tyrode buffer and exposed to UVB (20 kJ/m^2^) for 60 min. The fluorescence intensity was visualized and photographed under a fluorescence microscope (CTR 6000, Leica, Wetzlar, Germany) after the addition of 2% paraformaldehyde.

### 3.7. Statistical Analysis

Data were analyzed using the analysis of variance (ANOVA) test of the statistical package for the social sciences (SPSS). The statistical differences among groups were assessed by using Duncan’s multiple range tests. Differences were considered significant at *p* < 0.05.

## 4. Conclusions

In conclusion, this study determined that phlorotannin Fucofuroeckol-A derived from brown algal *Ecklonia stolonifera* Okamura possesses strong protective activity against UVB-induced allergic reaction. Its protective activity was revealed via scavenging ROS production, which might cause the inhibition of mast cell degranulation, histamine release, cytokine production, and intracellular Ca^2+^ elevation in UVB-exposed RBL-2H3 cells. Further study on the protective capability of Fucofuroeckol-A against UVB-induced allergic reaction on other mast cells as well as on an in vivo model will be performed to support its application as a novel cosmeceutical.
